# Unlocking the curative potential of gene transfer and editing for hematopoietic disorders

**DOI:** 10.1016/j.exphem.2026.105493

**Published:** 2026-08-14

**Authors:** Kohei Shiroshita, Anne Stolz, Camille Malouf, Vu L. Tran, Jingjing Li, Mitchell J. Weiss, Luigi Naldini

**Affiliations:** a San Raffaele Telethon Institute for Gene Therapy (SR-Tiget), IRCCS San Raffaele Scientific Institute, Milan, Italy; b Department of Stem Cell Biology, National Institute of Global Health and Medicine, Japan Institute for Health Security, Tokyo, Japan; c Japan Society for the Promotion of Science, Tokyo, Japan; d Department of Pediatrics, Dr von Hauner Children’s Hospital, University Hospital, Ludwig-Maximilians-University Munich, Munich, Germany; e Montreal Clinical Research Institute, Montreal, Canada; f Laboratory of Human Genetics of Infectious Diseases, University of Texas Southwestern Medical Center, Dallas, TX; g St Vincent’s Center for Applied Medical Research, Sydney, Australia; h School of Biomedical Engineering, University of New South Wales, Sydney, Australia; i School of Clinical Medicine, University of New South Wales, Sydney, Australia; j Department of Hematology, St. Jude Children’s Research Hospital, Memphis, TN

## Abstract

Hematopoietic stem and progenitor cells (HSPCs) gene therapy may transform the therapeutic landscape for inherited hematological disorders and already offers potentially curative options for some diseases, including inborn errors of immunity and *β*-hemoglobinopathies. Its continued success relies on further refinement of gene transfer technologies, gene-editing tools such as clustered regularly interspaced short palindromic repeats (CRISPR) and associated systems (CRISPR-Cas), and optimized *ex vivo* HSPC manipulation protocols that ensure robust, long-term engraftment and clonal diversity with reduced-toxicity, nongenotoxic conditioning strategies. Here, we reviewed recent developments and refinements in gene transfer and editing technologies for HSPCs, while also discussing the critical limitations and hurdles to clinical translation, as recently presented at the New Investigator Committee Gene Therapy webinar. Future directions must prioritize integrating technological innovation with the development of equitable and simplified models to reduce costs and ensure that these life-saving cellular therapies reach patients worldwide.

Gene therapy has emerged as a transformative approach, offering the potential to treat a wide range of genetic disorders by addressing their underlying causes. Historically, the concept of gene therapy referred primarily to gene addition or replacement, in which viral vectors were used to introduce a transgene into the genome to restore the function of a defective gene. With advances in genome engineering technologies, the scope of gene therapy has since expanded to include gene-editing strategies. The development of programmable nucleases, such as zinc finger and clustered regularly interspaced short palindromic repeats (CRISPR) systems, has enabled precise and targeted genome modifications, including correcting pathogenic mutations, disabling malfunctioning genes, or removing disease-causing sequences [1]. Specifically, CRISPR/CRISPR-associated systems (CRISPR-Cas) tools create targeted DNA double-strand breaks (DSBs), which can be repaired either by nonhomologous end joining (NHEJ) for gene disruption while introducing variable-sized deletions that are usually detrimental, or by homology-directed repair (HDR) via co-delivery of donor templates for precise correction [2]. Together, gene transfer and gene-editing approaches now comprise the broader landscape of modern gene therapy.

The path from concept to clinical application of gene therapy has been challenging. Early genome engineering efforts in the 1980s and 1990s focused on introducing genes into plants [3-5] and animals [6]. This was followed by the development of gene delivery systems, including replication-deficient retroviruses [7] and adeno-associated viruses (AAV) [8-10], which laid the foundation for therapeutic applications in humans. Initial clinical trials using retrovirus to introduce a gene into T cells in the early 1990s, such as those for adenosine deaminase (ADA) deficiency [11], demonstrated proof of concept but also revealed limitations, including transient efficacy when targeting T cells and continued dependence on enzyme replacement therapy. Subsequent trials targeting HSPCs and employing moderate conditioning achieved excellent and durable benefits in some immunodeficiencies, but other therapies revealed serious vector-related risks [12]. In particular, retroviral vector-based gene therapies for immunodeficiencies such as X-linked severe combined immunodeficiency (X-SCID) and Wiskott–Aldrich syndrome (WAS) caused leukemia in some patients, triggered by vector insertional activation of proto-oncogenes [13,14]. In addition, life-threatening immune responses to certain viral vectors, especially adenoviral vectors, highlighted the need for safer *in vivo* delivery systems [15]. Many of these early setbacks have been addressed through advancements in vector design, delivery methods, and gene-editing technologies. Regulatory bodies such as the US Food and Drug Administration (FDA) and the European Medicines Agency (EMA) have since approved several gene therapies. The first FDA approvals occurred in 2017, followed by EMA approvals in 2018. These included HSPC gene therapy for ADA-SCID (Strimvelis; Migliavacca et al. [16]), CAR-T-cell therapies for B-cell malignancies, and AAV-based treatments for inherited retinal diseases. In 2022 and 2023, additional gene therapies gained approval in both the United States and Europe, including AAV-based treatments for hemophilia A and B, as well as for Duchenne muscular dystrophy. Today, approved lentiviral HSPC gene therapies target conditions such as primary immunodeficiencies (WASs), hemoglobinopathies (lovo-cel; Kanter et al. [17]), lysosomal storage disorders such as adrenoleukodystrophy [18], and metachromatic leukodystrophy [19], and numerous others are undergoing preclinical or clinical evaluation. HSPC gene editing using CRISPR-Cas9 also reached its first market approval for β-hemoglobinopathies (exa-cel) in 2023 [20]. Although canonical CRISPR-Cas systems function by creating targeted DSBs at specific genomic loci, newer modalities such as base editors (BEs) and prime editors (PEs) enable precise nucleotide substitutions or short insertions without intentionally generating DSBs [21]. Approved or late-stage HSPC gene therapy products are summarized in Table 1.

As the field advances, genome editing remains a particularly promising technique with the potential to redefine the treatment landscape for inherited hematopoietic disorders. For these therapies to be successful, it is crucial to modify bone marrow–repopulating, bona fide hematopoietic stem cells (HSCs), which represent a small fraction of the HSPC population. At the same time, significant challenges remain, including optimizing the delivery of genome editors to HSPCs, minimizing genotoxicity, and preserving the long-term functionality of genetically modified HSPCs. In May 2025, the International Society for Experimental Hematology (ISEH)’s New Investigator Committee hosted a webinar featuring HSC gene therapies (available to view online at https://my.iseh.org/general/custom.asp?page=Webinars). Here, we summarized discussions led by HSC gene therapy experts Prof Luigi Naldini and Prof Mitchell Weiss, including key principles, challenges, and examples of their recent strategies and applications.

## PRINCIPLES OF HSPC GENE THERAPY

The gene therapy approach is built on the foundation of stem cell transplantation. In this method, autologous HSPCs are harvested from the patient and then modified *ex vivo* via gene transfer or gene editing before being reinfused (Figure 1A). Before infusion, the patient undergoes myelotoxic conditioning to empty stem cell niches in the bone marrow and promote engraftment of the corrected cells, aiming to establish a potentially lifelong supply of gene-corrected or gene-modified cells across all hematopoietic lineages.

A foremost challenge in HSPC gene therapy is achieving efficient genetic modification without compromising long-term repopulating capacity. This must be balanced with overcoming innate inflammatory barriers, ensuring safety from genotoxicity, and preserving physiological gene expression patterns. First, the gene therapy protocol must be optimized to ensure that a sufficient number of HSCs are successfully modified before infusion, as low modification rates, such as those observed in sickle cell disease trials, can limit durable engraftment. Second, it is crucial to overcome innate inflammatory cellular barriers that can reduce the transduction efficiency of viral vectors or trigger adverse responses; for instance, HSC inflammatory sensing that hampers lentiviral vector transduction [22], or reduced engraftment potential mediated by activation of the DNA DSB–p53 axis [23]. Third, preventing genotoxicity risks remains crucial, most importantly to minimize unintended activation of oncogenes or inactivation of tumor suppressor genes by viral vector insertion or off-target genome editing, as discussed in a later section.

Despite these challenges, the field has developed successful, clinically approved treatments for genetic disorders, including primary immunodeficiencies, hemoglobinopathies, and metabolic syndromes.

Metachromatic leukodystrophy (MLD) is an ultrarare, severe lysosomal storage disease caused by arylsulfatase A (ARSA) deficiency, leading to neurological impairment, including motor and cognitive disorders [24]. For early-onset MLD, a devastating demyelinating disease leading to early death, early treatment, ideally before symptom onset, can halt disease onset and evolution of the disease. Previous studies demonstrated that HSCs exhibit plasticity, enabling their differentiation into cells of several nonhematopoietic organs, including microglia in the central nervous system (CNS) [25,26]. This suggests that HSCs can be a therapeutic target for some CNS diseases, and HSPC gene therapy is poised to become a preferred alternative, as it eliminates the need for a matching donor and avoids immune-related complications such as rejection or graft-versus-host disease. Importantly, overexpression of therapeutic enzymes can enhance cross-correction, including in the brain tissue in the case of MLD. The San Raffaele Telethon Institute for Gene Therapy (SR-Tiget) group showed proof of concept of lentiviral-based HSPC gene therapy for MLD in a preclinical mouse model [27] and then conducted gene therapy clinical trials using lentiviral vectors carrying the ARSA gene [28], in which patients were monitored for >7 years [19]. Vector insertion site analysis revealed stable engraftment, averaging 1–2 vector copies per genome. In a related study led by Eugenio Montini’s group at SR-Tiget involving patients enrolled in trials for MLD, WAS, and *β*-thalassemia, researchers showed highly polyclonal grafts with up to thousands of distinct, nondominant clones, with each contributing <1% [29]. They also quantified the number of long-term repopulating HSCs in these patients and found that there are between several thousands and up to 20,000 active lentiviral-transduced HSPC clones over the long term. These levels are comparable to those observed after allogeneic HSPC transplantation [30], which highlights strong polyclonal engraftment and future resilience. Variations in these numbers likely reflect differences in conditioning regimens and the quality of stem cell sources. These studies demonstrated that lentiviral HSPC gene therapy can achieve durable engraftment and show meaningful therapeutic efficacy.

The use of CRISPR-mediated HDR is limited by its low efficiency in HSCs. Naldini’s team has improved HDR efficiency in human HSCs by reducing p53-mediated DNA damage responses (Figure 1B). In preclinical studies, they transiently inhibited the p53 response by co-delivering, together with the editor, a dominant-negative form of p53 [23]. By transiently inhibiting p53 during editing, they preserved engraftment and repopulating clonal diversity in xenotransplant models [31]. Edited HSCs demonstrated robust multilineage reconstitution and serial transplantation capacity, demonstrating true long-term repopulating potential. The efficiency of HDR editing can be further optimized by the choice of repair templates such as adeno-associated virus or integration-defective lentiviral vectors [2]. Delivery methods, such as lipid nanoparticle-based systems, are considered less disruptive than electroporation of ribonucleoprotein complexes and are under refinement to minimize cell stress [32]. The outcomes of edits are highly heterogeneous due to the mixture of different DNA repair mechanisms, including HDR, NHEJ, and microhomology-mediated end joining [33].

## CHALLENGES OF HSPC GENE THERAPY

Major challenges of HSPC gene therapy are concentrated in three areas: (1) vector integration and enhancer-mediated genotoxicity, (2) nuclease-related undesired outcomes, and (3) product-level risks. These points are summarized in Table 2.

### Vector Integration and Enhancer-Mediated Genotoxicity

One of the most critical issues in the early development of gene therapy was the genotoxicity associated with *γ*-retroviral vectors [53]. Early trials using *γ*-retroviral vectors demonstrated a risk of insertional oncogenesis, with several cases of leukemia attributed to strong promoters and enhancers in the vector long-terminal repeats (LTRs). In contrast, lentiviral vectors largely avoid this risk, as they are designed with self-inactivating LTRs, meaning that they do not carry any transcriptional elements in the LTRs after insertion, and use moderate cellular promoters to drive transgene expression. However, even self-inactivating lentiviral vectors can pose risks if equipped with powerful promoters, underscoring the importance of vector design and integration patterns. Despite this, vector insertion can still occasionally disrupt genes or regulatory elements, leading to rare but potentially harmful aberrant transcription. In a clinical trial of lentiviral vector-mediated gene therapy for X-linked adrenoleukodystrophy, a significant fraction of treated patients developed myeloid malignancies due to vector integrations near the *MECOM* or *PRDM1* oncogenes. The serious toxicity was most likely driven by the unique vector design, which included a strong *γ*-retroviral–derived promoter [54]. Thus, promoter selection for a lentiviral gene therapy vector should balance sufficient transgene expression to achieve therapeutic efficacy with the risk of genotoxicity from inadvertent insertional activation of oncogenes [55]. Moreover, neuroepithelial tumor development due to AAV integration after gene therapy has been observed [56]. Concatemeric insertions of AAV genomes and their fragments have been documented [2], and strategies to mitigate this issue have also been reported [36]. Thus, assessing vector integration sites is crucial for understanding vector-related genotoxicity [34,35].

### Nuclease-Related Editing Outcomes

DNA DSBs induced by nucleases such as Cas9 are repaired by multiple DNA repair pathways, generating heterogeneous editing outcomes.

Another major problem of nuclease-related approaches is off-target editing [57]. Early studies relied on in silico analysis or cellular assays to predict and validate potential off-target loci [37-41]. Small insertions or deletions can be detected by Sanger sequencing methods such as TIDE [58] and ICE [42], or direct amplicon sequencing using next-generation sequencing (NGS). Although selecting high-specificity guide RNAs (gRNAs), high-fidelity Cas9 [46], nickase Cas9 [59], and DSB-free editors (BEs and PEs) are important strategies to reduce off-target effects, these cannot be eliminated [60].

Another hurdle is the potential generation of structural variants (SVs), including large deletions, chromosomal translocations, inversions, and duplications. Although karyotyping or fluorescence in situ hybridization (FISH) is widely used in the clinical setting, these conventional assays often lack sufficient sensitivity to detect low-level SVs and may underestimate their frequency. Recent technological advancements enable the precise detection of nuclease-induced SVs with high sensitivity. Drop-off digital PCR assay and optical genome mapping are useful platforms to detect large deletions or genome-wide rearrangements [33]. CAST-seq is a pioneering technology for detecting chromosomal translocations (sensitivity ≥ 0.5%), as well as on-target genomic aberrations [44]. To detect translocations more sensitively, high-resolution CAST-seq was developed by combining an NHEJ repair inhibitor [61]. CLEAR-time digital PCR can comprehensively assess genomic aberrations and is useful for understanding the kinetics of DNA repair outcomes [45]. These platforms are widely used to investigate editing at clinically relevant loci [62-65] and shed light on genomic integrity of cells treated with BEs and PEs [60,66].

Conventional DNA amplicon sequencing by NGS cannot capture large deletions or translocations and introduces a risk of PCR bias. Long-read sequencing technologies, such as Oxford Nanopore or PacBio, facilitate long-range sequencing of native DNA or RNA for detecting SVs without PCR amplification. The DNA-dependent protein kinase catalytic subunit (DNA-PKcs) inhibitor AZD7648, which enhances HDR repair by inhibiting NHEJ machinery, not only increases HDR efficiency but also increases kilobase-scale large deletions [67]. Although an HDR template can mitigate translocation frequency, it cannot reduce large deletions [61].

Impaired genomic integrity after gene editing raises concerns regarding the risk of tumorigenesis or the disruption of HSPC function. Thus, genomic integrity must be carefully evaluated during clinical translation. Recent studies have revealed that targeting proliferation stress [47] or senescence and inflammatory responses [48] during CRISPR-Cas9 editing represents potential approaches to ameliorate genotoxicity. Purifying H DR-edited HSPCs based on transient expression of a selector could also be a reasonable strategy [43].

### Product-Level Risks (Clonal Expansion and Clonal Hematopoiesis)

Clinically important genotoxic events usually manifest first as clonal expansions rather than immediate transformation. Additional factors, such as disease-associated inflammation, hematopoietic stress, and product diversity, can influence whether an expanding clone outcompetes others. There is also evidence that pre-existing mutations may be selected during *ex vivo* culture and post-transplantation, promoting clonal dominance [30,68]. Thus, it is important to monitor clonal architectures and perform close surveillance for long-term malignancies after gene therapies by using emerging clonal tracking technologies [49,-51,69].

There are still hurdles to overcome in future studies. *Ex vivo* manipulation can be detrimental to HSCs, which are difficult to expand without promoting differentiation. Chemotherapy-based conditioning regimens still require refinement to improve safety. Long-term follow-up is critical to monitor safety, efficacy, and polyclonal engraftment.

## THERAPEUTIC MODULATION OF GLOBIN GENES FOR *β*-HEMOGLOBINOPATHIES

The development of gene therapies for *β*-hemoglobinopathies clearly exemplifies how decades of fundamental research on globin gene regulation have paved the way for effective therapeutic interventions. Dr Mitchell Weiss is a pioneer in the field of *β*-hemoglobinopathies and red blood cell development. His work has focused on understanding and treating sickle cell disease and *β*-thalassemia, which are associated with multiple mutations in the *HBB* gene encoding adult-type β-globin. *β*-globin is an essential part of hemoglobin, the red blood cell oxygen carrier.

Hemoglobin is a heterotetramer composed of globin genes that are developmentally regulated: early embryos will produce epsilon- and zeta-globin chains, fetuses will produce fetal hemoglobin (HbF) consisting of two alpha and two gamma chains, and adults will produce adult hemoglobin (HbA) formed by two alpha and two beta chains. This process remains highly dynamic after birth, with most of the HbF being replaced by HbA within the first year of life. The main role of all types of hemoglobin is to carry oxygen to our different cells and tissues. Dr Weiss presented a case study of a newborn baby girl who presented with cyanosis (blue-tinged skin color) and low blood oxygen levels, a condition that was also seen in her father at birth and cleared itself a few days later. Her hematologists identified a hereditary cause for this “Blue Baby” syndrome. Specifically, the patient had a specific *γ*-globin variant (*HBG2* p. V67M) with an amino acid substitution (valine to methionine) in the globin heme pocket that inhibits oxygen binding to HbF. Similar to her father, the baby girl’s cyanosis resolved within a few weeks after birth, and her blood oxygen levels normalized, coinciding with the physiological switch from HbF to HbA. By convention, the mutation was named “Hemoglobin Toms River,” after the city in which the variant was identified [70]. This case study illustrates perfectly how a severe hemoglobinopathy can be reversed with the coordinated expression of developmentally regulated genes.

The onset of *β*-thalassemia and sickle cell disease in patients also coincides with the perinatal *γ*-to-*β* switch (HbF to HbA). In contrast to the cyanotic Hemoglobin Toms River patient, hemoglobinopathy patients become symptomatic after birth when normal adult hemoglobin expression fails to develop. *β*-hemoglobinopathy patients with high HbF levels have less-severe symptoms and are more clinically manageable [71,72]. Individuals with a benign condition termed “Hereditary Persistence of Fetal Hemoglobin” (HPFH) express higher levels of HbF throughout life. Individuals who co-inherit HPFH and *β*-hemoglobinopathy frequently exhibit reduced or absent symptoms. This clinical observation has inspired gene therapies for *β*-hemoglobinopathy. Numerous ongoing gene therapy trials for *β*-hemoglobinopathies are based on HbF induction.

In 2026, a successful “*ex vivo*” gene therapy for sickle cell disease or *β*-thalassemia requires five main steps that are all critical: informed consent, isolation of HSPCs, genetic manipulation of HSPCs to either induce HbF expression in erythroid cells or replace the defective *HBB* gene, bone marrow conditioning, and transplantation of modified, functional HSCs back into the patient. The precise genomic sites targeted by genetic manipulations to induce HbF rely on information obtained from basic research over the past decades, especially in the 1980s and 1990s during the identification of hereditary persistence of HbF variants [73-78]. Many of these variants are located in the *γ*-globin gene promoter, and their presence partially reverses the γ-to *γ*-globin gene switch, thereby maintaining therapeutic levels of HbF throughout life [79]. Genetic variants associated with HbF expression also map to the erythroid-specific *BCL11A* gene, which encodes a transcriptional repressor of the *γ*-globin genes [80]. One approach used for gene therapy has been to exploit the Cas9 technology to disrupt the *BCL11A* binding motif in the *γ*-globin promoter (no binding of *BCL11A*, specific cellular effect) (Figure 2A) or to disrupt the *BCL11A* erythroid enhancer (eliminates *BCL11A* expression in erythroid cells) in patient HSPCs (Figure 2B). These manipulations will induce γ-globin expression and HbF production in erythroid cells derived from the modified HSPCs. Both approaches have worked extremely well for patients with β-thalassemia or sickle cell disease [81-86]. Further studies on those two approaches (disrupting BCL11A binding motif vs. decreasing BCL11A expression) have shown that the disruption of BCL11A expression by Cas9 leads to increased apoptosis of CD34^+^-derived erythroid cells in vitro, and a significant dropout of BCL11A-edited erythroblasts after xenotransplantation into mice, which has been linked to the altered expression of BCL11A-regulated genes [87,88]. Although these observations point to potentially unappreciated roles for *BCL11A* in erythropoiesis beyond HbF expression, patients treated with exa-cel exhibit remarkable clinical outcomes, suggesting that this theoretical risk is outweighed by potential benefits [81,89].

More recently, scientists have used a technology called adenine base editing (BE), which works by converting A:T base pairs to G:C. Using this technology [90], it has been possible to recapitulate three forms of HPFH that create new transcription factor–binding sites in the *γ*-globin gene promoter, thereby reactivating its expression in adult red blood cells. For example, the −175 A>G variant creates a composite GATA1-TAL1 motif that stimulates interaction of γ-globin promoters with the locus control region, a powerful upstream enhancer (Figure 2C) [91]. Recent results from two clinical trials demonstrated the safety and efficacy of adenine base editor targeting the *γ*-globin promoter to induce HbF expression in severe sickle cell disease [92] and γ-thalassemia [93] (Table 3).

These scientific advances are significantly improving outcomes for patients who previously faced limited life expectancy. They can now pursue long-term projects that were not possible before these therapies.

## CONCLUSIONS AND FUTURE DIRECTIONS

The work presented by Drs Luigi Naldini and Mitchell Weiss highlights the profound development and therapeutic potential of HSPC gene therapy, offering transformative strategies for treating human hematopoietic disorders.

Although these advances have brought the field closer to potentially curative options, several critical challenges must be solved before these therapies can be broadly implemented (see Outstanding challenges box). A fundamental issue is the difficulty of securing sufficient numbers of HSPCs, particularly from patients with conditions like sickle cell disease, which often necessitates multiple, burdensome mobilization procedures [94]. To overcome this limitation, the development of novel, more efficient mobilization reagents such as Motixafortide is needed [95]. Another long-term possibility is to use induced pluripotent stem cells (iPSCs) as an alternative donor source. New culture protocols for iPSCs have generated an HSC-like population that can engraft mouse bone marrow in xenotransplantation studies [96]. Thus, it may eventually be possible to generate autologous, gene-corrected HSPCs from iPSCs of patients with blood disorders, a strategy that will be tested in the future. Equally crucial is mitigating the toxicity of the current bone marrow conditioning regimens to reduce long-term risks such as secondary malignancies and organ damage. Of note, many patients with sickle cell disease and *β*-thalassemia have compromised organ function and therefore cannot tolerate toxic alkylating agents like busulfan. Thus, researchers are actively exploring gentler alternatives for bone marrow conditioning, including immune-depletion of host HSPCs with anti-CD45 [97], anti-c-Kit [98], or anti-MPL monoclonal antibodies [99], and HSPC mobilization-based, chemotherapy-free conditioning [100] in preclinical models and clinical trials. Another major obstacle is to improve the global accessibility of transformative gene therapies for patients with *β*-hemoglobinopathies. Since the majority of patients live in low-income countries that lack the infrastructure for sophisticated cellular therapy [101], there is a pressing need to develop lower-cost, simpler therapeutic methods.

Furthermore, the sustainability of gene therapy is challenged by complex regulations, high manufacturing costs, and economic pressures where traditional biopharma models struggle to ensure a return on investment, ultimately jeopardizing market viability and patient access to these life-saving treatments [102-104]. In particular, the high economical cost represents a major barrier for the commercial development of *ex vivo* gene therapy products. For instance, reni-cel, an autologous HSPC product edited with AsCas12a at the fetal *γ*-globin gene promoters [105], demonstrated excellent efficacy and safety in clinical trials [92,93]. However, economic reasons led the biotech company to discontinue its development [106]. Balancing cost, benefit, and equitable access represents the overarching challenges for the future of the field [107]. To expand accessibility of gene therapy, simplified manufacturing, regional GMP centers, reduced-toxicity conditioning, shorter *ex vivo* culture, standardized potency assays, and *in vivo* HSPC-targeted delivery platforms are fundamental.

*In vivo* gene therapy is rapidly emerging as a transformative approach for treating hematologic disorders, offering the potential to correct disease-causing mutations directly in patients’ tissues without *ex vivo* manipulation (Figure 1C). Recent studies have shown that lipid nanoparticles (LNPs) [108,109] or virus-like particles [110,111] are powerful tools for in vivo gene delivery, particularly when combined with targeting HSPCs via enriched cell surface antigens, such as CD117 [108] or CD133 [111]. Additionally, systemic lentiviral vector administration enabled *in vivo* gene delivery in newborn mice and successfully treated mouse models of several immunodeficiencies, suggesting that circulating HSPCs are promising targets for *in vivo* editing [112]. Regarding gene-editing platforms, BEs and PEs are emerging tools for *in vivo* HSPC editing [113-115]. Although BEs/PEs have limitations in terms of gene-correction length, which is smaller than HDR-based gene correction, these methods are more suitable for in vivo HSPC editing because they overcome the cell-cycle dependency of the HDR machinery. Recently, a patient with carbamoyl phosphate synthetase 1 (CPS-1) deficiency, an ultrarare hepatic inborn error of metabolism, was successfully treated within 8 months of diagnosis using hepatocyte-targeting LNPs containing BE mRNA and a localizing guide RNA [116]. These studies highlight the potential for treating rare diseases, enabling rapid therapy, and underscore the need for personalized gene therapy in the future.

Finally, in addition to following clinical endpoints such as transfusion independence or freedom from vaso-occlusive crises, gene therapy studies must analyze comprehensive patient-centered outcomes, including the preservation of fertility, the maintenance or recovery of organ function, and overall health-related quality of life— to ensure the long-term well-being of patients undergoing *ex vivo* gene and cell therapies.

The next stage of HSPC gene therapy will depend not only on improved genome engineering technologies but also on safer bone marrow conditioning, more efficient manufacturing, robust long-term surveillance, and health-system models that broaden access beyond a small number of high-income countries.

## Figures and Tables

**Figure 1:**
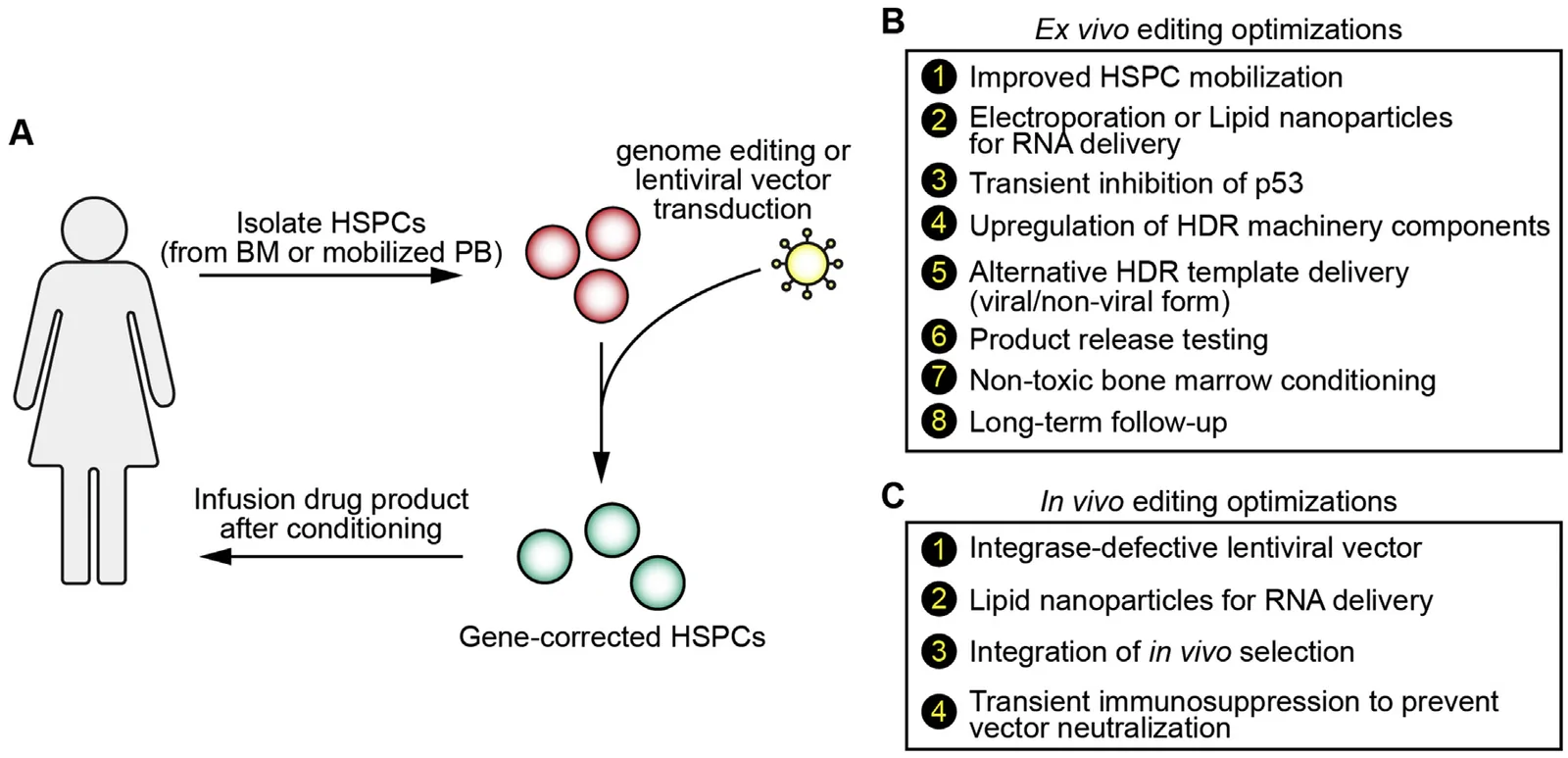
Optimizations to enhance gene therapy efficacy. (**A**) General process of *ex vivo* gene therapy using both CRISPR-Cas9 and gene addition/replacement strategies, and an exogenous DNA template to correct disease-causing variants in primary HSPCs isolated from patients. (**B**) Strategies to improve *ex vivo* HSPC gene therapy include optimizing HSPC mobilization, delivery methods of editing reagents (electroporation versus lipid nanoparticles), transient inhibition of p53, and upregulating components of HDR machinery, alternative template delivery (viral or nonviral HDR templates), product release testing, nongenotoxic bone marrow conditioning, and long-term follow-up. (**C**) Strategies to optimize *in vivo* HSPC gene therapy include integrase-defective lentiviral vector, alternative template delivery, lipid nanoparticle-based system to replace electroporation, integration of *in vivo* selection, and transient immunosuppression to prevent vector neutralization. BM=bone marrow; HDR=homology-directed repair; HSPCs=hematopoietic stem and progenitor cells; PB=peripheral blood *HDR*=Homology-directed repair; *HSPCs*=hematopoietic stem and progenitor cells.

**Figure 2:**
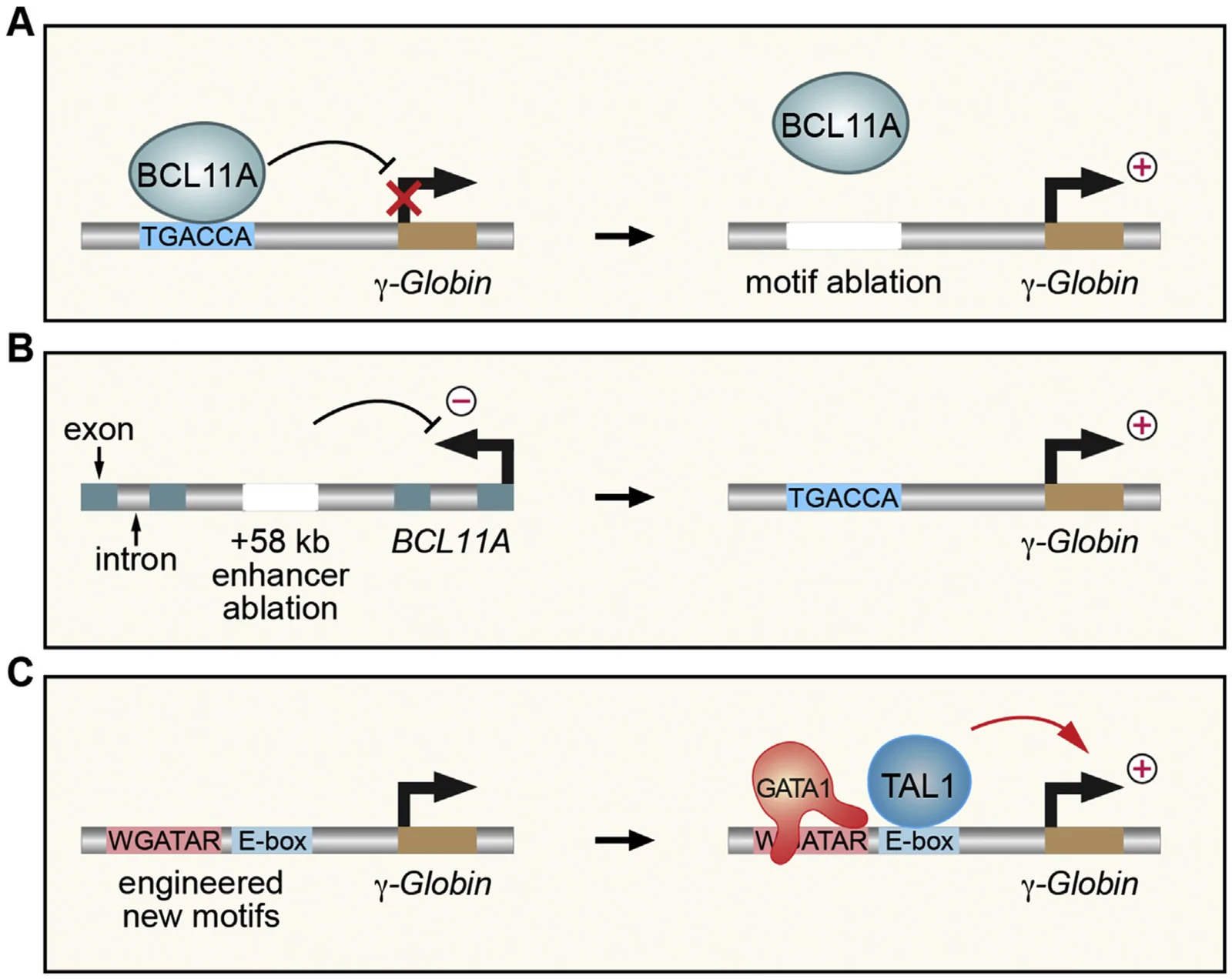
Different strategies to re-activate fetal hemoglobin for therapeutic applications. (**A**) Ablation of the binding motif of the negative regulator BCL11A in the *γ*-globin gene promoters disrupts BCL11A’s ability to repress *γ*-globin gene expression. (**B**) Ablation of the BCL11A erythroid enhancer +58 kb downstream of the *BCL11A* transcription start site reduces *BCL11A* expression. (**C**) Creating new binding motifs for transcription factors such as GATA1 and TAL1 to activate *γ*-globin gene expression.

**Table 1:** Approved and late-stage *ex vivo* HSPC gene therapy products

Brand name (generic name)	Disease indication	Vector/editing strategy	Conditioning requirement	Regulatory region/status	Major safety or follow-up issue
Strimvelis (Otilimabogene autotemcel)	Adenosine deaminase –deficient severe immunodeficiency (ADA-SCID)	*γ*-Retroviral vector	Mild conditioning (Busulfan or Melphalan)	EMA (approved in 2016)	Insertional mutagenesis Monitoring for clonal dominance
Libmeldy/Lenmeldy (atidarsagene-autotemcel)	Metachromatic leukodystrophy (MLD)	SIN-LV	Myeloablative conditioning (Busulfan)	EMA (2020) FDA (2024 as Lenmeldy)	Long-term insertional site tracking Immune responses to enzyme
Skysona (elivaldogene autotemcel: eli-cel)	Cerebral Adrenoleukodystrophy (CALD)	SIN-LV	Myeloablative conditioning	FDA (approved 2022)	Clonal dominance Insertional oncogenesis (MECOM activation)
Zynteglo (betibeglogene autotemcel: beti-cel)	*β*-Thalassemia	SIN-LV	Myeloablative conditioning (Busulfan)	FDA (2022) EMA (previously approved, withdrawn commercially)	Insertional site monitoring Transfusion independence durability
Lyfgenia (lovotibeglogene autotemcel: lovo-cel)	Sickle cell Disease (SCD)	SIN-LV (with modifications)	Myeloablative conditioning (Busulfan)	FDA (approved 2023)	Insertional genotoxicity monitoring Monitoring for hematologic malignancy
Casgevy (exagamglogene autotemcel: exa-cel)	*β*-Thalassemia / SCD	CRISPR-Cas9 (BCL11A enhancer disruption)	Myeloablative conditioning (Busulfan)	FDA, EMA, MHRA (approved 2023–2024)	Off-target cleavage Large deletions/structural variants, p53-mediated clonal stress
Waskyra (etuvetidigene autotemcel)	Wiskott–Aldrich syndrome (WAS)	SIN-LV	Myeloablative conditioning	EMA/FDA (approved 2025)	Long-term immune reconstitution and insertion-site safety
Kresladi (marnetegragene autotemcel)	Leukocyte Adhesion Deficiency-I (LAD-I)	SIN-LV	Reduced-intensity/Myeloablative conditioning	Late-stage Regulatory review (FDA approved in 2024 under name Kebilidi)	Engraftment durability Vector persistence

*EMA*=European Medicines Agency; *FDA*=Food and Drug Administration; *MHRA*=Medicines and Health care products Regulatory Agency, *SIN-LV*=self-inactivating lentiviral vector.

**Table 2:** Type of genotoxicity and its evaluation methods

Type of genotoxicity/risk		Mechanism	Assessment platform	Prevention and mitigation (potential)
Vector integration and enhancer-mediated genotoxicity	Vector-mediated insertional mutagenesis	Transactivation of proto-oncogenes by viral LTRs or insertional disruption of tumor suppressor genes	VISPA (LV) [34] RAAVioli (AAV)^35^	Integrase-deficient LV (IDLV) Choice nonviral delivery (Cas9, HDR templates) Mitigating concatemeric integration [36] (AAV)
Nuclease-related editing outcomes	CRISPR off-target mutations	Sequence-dependent unintended DSBs and error-prone repair	Cell-based methods: GUIDE-seq [37], DISCOVER-seq [38] In vitro assay: CIRCLE-seq [39], Digenome-seq [40], CHANGE-seq [41]	High-fidelity Cas9 variants [42] Rigorous gRNA in silico design and validation
	On-target large deletions Structural variants (SVs)	Extensive end-processing, fragment integrations, or large deletions around the Cas9 cut site	Karyotyping FISH Copy number variation by ddPCR [33,43] CAST-seq [44] CLEAR-time dPCR [45] Long-read sequencing [33,43] Optical genome mapping [33] Single-cell DNA sequencing (e.g., Tapestri)	Nickase Cas9 variants [46] Use non-DSB editors (base/prime editor) Mitigation of culture-induced genotoxic stress [47,48] Selection of correctly edited cells [43]
	Chromosomal translocations	Simultaneous DSBs at multiple loci (on- and off-targets) leading to aberrant re-joining		Transient delivery of RNP to limit exposure time gRNA optimization Mitigation of culture-induced genotoxic stress [47,48] Selection of correctly edited cells [43]
Product-level risks	Clonal expansion and clonal hematopoiesis	*Ex vivo* culture stress, inflammatory microenvironments, or selection of pre-existing/new mutant clones (e.g., *TP53*)	Integration site analysis (LV) [34] Barcoding HDR template (AAV): BAR-seq [49], TRACER-seq [50] Mitochondria clonal tracking: ReDEEM [51], MAESTER [52] Single-cell clonal tracking: EPI-Clone [52]	Identification of pre-existing mutations before gene therapy Monitoring clonal diversity after gene therapy

*AAV*=Adeno-associated virus; *DSB*=double-strand break; *HDR*=homology-directed repair; *LV*=lentiviral vector; *LTR*=long-terminal repeats; *RNP*=ribonucleoprotein.

**Table 3:** Different gene-editing approaches targeting *β*-hemoglobinopathies

Evaluation axis/ Comparison items	DSB-dependent editing			DSB-free editing (base editing)	
	Enhancer	Promoter		Promoter	CDS
Target locus	Erythroid-specific BCL11A enhancer	HGB1/2 promoter regions		HBG1/2 promoter regions	HBG1/HGB2 gene
Editing mechanism	CRISPR-Cas9 mediated disruption of erythroid-specific BCL11A enhancer	CRISPR-Cas12a (Cpf1) mediated DSB or modification of repressor binding site	CRISPR-Cas9 mediated DSB or modification of repressor binding site	Adenine base editor (ABE) mediated precise bases conversion (A-to-G) to introduce mutations of Hereditary Persistence of Fetal Hemoglobin (HPFH)	Target bases conversion to correct disease-causing mutations
Expected HbF induction	High (Indirect yet potent HbF re-induction via erythroid BCL11A suppression)	High (Direct and efficient induction of *γ*-globin expression)		Intermediate to High (Precise introduction of HPFH-like mutation)	Not applicable (Direct correction of abnormal HbS to normal/benign variant)
Potential genotoxicity	Off-target cuts large deletions, translocations, structural variants (SVs) p53-mediated stress			Potential RNA/DNA off-target effects or bystander edits Lower DSB-associated structural variant risks	
Clinical maturity	Commercially approved most extensive clinical data	Early to mid-stage clinical trials		Preclinical to first-in-human trials	Preclinical to first-in-human trials
Clinical trial (target disease) Drug product	CLIMB THAL-111/SCD-121 trial (TDT/SCD) DP: exa-cel	EDIT-301 (SCD/TDT)^a^ DP: reni-cel	SAGES1 (SCD), NCT06506461	BEAM-101 (SCD/TDT)	BEAM-102 (SCD)
Long-term follow-up requirements	Up to 15 years follow-up of clonal tracking, insertion/variant monitoring, and malignancy watch	Long-term clonal dynamics and safety monitoring common to gene editing	Long-term clonal dynamics and safety monitoring common to gene editing	Long-term tracking of potential off-target/bystander editing outcomes and clonal dynamics	Long-term tracking of potential off-target/bystander editing outcomes and clonal dynamics
Manufacturing complexity	High (HSPC collection, optimal delivery of editor proteins/mRNA (electroporation/lipid nanoparticle), *ex vivo* culture, and strict quality control)				

*CDS*=coding sequence; *DSB*=double-strand break; *DP*=drug product; *SCD*=sickle cell disease; *TDT*=transfusion-dependent thalassemia.

a This trial was terminated for business reasons.

## HIGHLIGHTS

- Gene editing corrects genetic mutations in hematopoietic disorders
- Gene editing enables precise engineering for therapeutic applications
- Hematopoietic stem and progenitor cells (HSPCs) gene therapy using lentiviral vectors (LVs) shows curative potential
- Several successful clinical trials using LVs have resulted in approved therapies
- Key hurdles include product safety, complex logistics, and equitable global access

## Outstanding challenges box

1. HSPC collection and mobilization.
2. Preservation of long-term repopulating capacity.
3. Conditioning toxicity.
4. Genotoxicity and on-target structural variants.
5. Product manufacturing.
6. Long-term follow-up.
7. *In vivo* HSPC targeting gene therapy.
8. Global access.
